# Complete genome sequence of *Bacillus velezensis* B31, a potential biocontrol agent with ability of tolerance to fusaric acid and antagonism against *Fusarium oxysporum*

**DOI:** 10.1128/mra.00955-23

**Published:** 2024-01-18

**Authors:** Zhenhe Su, Xiaomeng Liu, Lihong Dong, Qinggang Guo, Shezeng Li, Ping Ma

**Affiliations:** 1 Institute of Plant Protection, Hebei Academy of Agriculture and Forestry Sciences, Integrated Pest Management Innovative Center of Hebei Province, Key Laboratory of IPM on Crops in Northern Region of North China, Ministry of Agriculture and Rural Affairs of China, Baoding, China; The University of Arizona, Tucson, Arizona, USA

**Keywords:** complete genome, *Bacillus velezensis*, tolerante to fusaric acid, *Fusarium oxysporum*

## Abstract

*Bacillus velezensis* B31 is tolerant to fusaric acid, exhibits antagonism against *Fusarium oxysporum*, and has an excellent control effect on tomato fusarium wilt. Here, we present the complete genome sequence of B31, which contains 4,056,755 bp DNA with a G + C ratio of 46.39%. The genome has 3,838 protein-coding genes.

## ANNOUNCEMENT

*Bacillus* species are Gram-positive, endospore-forming, aerobic, or facultatively anaerobic bacteria that are ubiquitous in the nature environment. They are also known for their antagonistic ability against diverse phytopathogens (1). In our previous study, we established a method for screening biocontrol resources against fusarium wilt based on tolerance to fusaric acid (FA) and antagonism against *Fusarium oxysporum* (2). We obtained strain B31 from tomato rhizosphere soil, which successfully controlled tomato fusarium wilt and exhibited increased tolerance to both *F. oxysporum* and its metabolites, including FA at 20 µg/mL, compared with *B. velezensis* strain FZB42 (2). Strain B31 has been identified *Bacillus velezensis* based on multigene genealogies of *gyrB*, *rpoB*, and *rpoC* (2). *B. velezensis* B31 is available at the China General Microbiological Culture Collection Center (CGMCC) with the CGMCC No. 24599.

Strain B31 was recovered on lysogeny broth (LB) agar from a −80°C stock supplemented with 10% glycerol, lined onto LB agar with sterilized toothpick, and then incubated at 30°C for 1 day till most bacterial colonies emerged. One random colony was transferred to 5 mL LB medium and incubated at 30°C with shaking at 180 rpm overnight. For extracting DNA, 2 mL aliquots of strain B31 was centrifuged and collected the precipitate, then lysed by lysozyme and protease, the purified DNA was extracted using Tiangen genomic DNA isolation kit (Tiangen, China), following the manufacturer’s recommended protocol. The B31 genome from the same DNA was sequenced using the Illumina Hiseq + PacBio sequencing system at Majorbio Biotech Co., Ltd. (Majorbio, China). The DNA was fragmented and prepared using a Covaris ultrasonic disruptor and the NEBNext Ultra DNA Library Prep Kit, then sequenced on the Illumina HighSeq 4000 platform. This resulted in over 4,234,558*2 reads and 1,278,836,516 bp sequenced DNA. The original reads from the PE library were aligned to the assembly sequence using SOAP software (v2.04) to obtain the base depth (3). Prior to PacBio sequencing, genomic DNA was fragmented into 8–10k pieces, and the ends of these fragments were connected to form an SMRT Bell. This resulted in over 487,211 PacBio reads with 4,942,317,722 bp sequenced DNA and an N50 of 10,144 bp. The third-generation sequence assembly was performed using the Unicycler assembly software (v0.4.8) to return a circular chromosome (4), and the sequence correction was performed with Pilonjin software (v1.22) during the assembly process (5). The NCBI Prokaryotic Genomes Automatic Annotation Pipeline was used for genome annotation, which employed GeneMark, Glimmer, and tRNAs-can-SE tools to annotate the genome of strain B31 (6). All software used default parameters unless otherwise indicated. Finally, the genome of strain B31 was deposited in the GenBank database by the National Center for Biotechnology Information (NCBI).

The genome is a circle chromosome with a length of 4,056,755 bp base pairs with GC content of 46.39%. There were a total of 4,024, including 3,838 protein-coding genes, 86 tRNAs, 27 rRNAs, 5 ncRNAs, and 68 pseudo genes. The genome will provide molecular contribution on illustrating mechanism of inhibiting *F. oxysporum* of *B. velezensis* B31.

## Data Availability

This Whole-Genome Shotgun project has been deposited in DDBJ/ENA/GenBank under accession no. PRJNA974370. The version described in this paper is the first version, PRJNA974370. The assembly sequence can be accessed through https://www.ncbi.nlm.nih.gov/assembly/GCF_030127405.1/?shouldredirect=false. The Genbank accession for this genome was CP126226.1. Both Illumina (SRX22619941) and PacBio (SRX22575911) reads have been uploaded to the SRA database.
